# The Gut–Brain–Immune Axis: Role of Microbiota Dysbiosis and Autonomic Nervous System in Infectious Diseases

**DOI:** 10.7759/cureus.108276

**Published:** 2026-05-04

**Authors:** Shruti Tiwari, Uprinder Kaur, Narinder Kaur, Waqas Alauddin, Sayali Khairnar, Rosy Bala, Vipasha Kaushal, Mohit Mishra

**Affiliations:** 1 Microbiology, Maharishi Markandeshwar Institute of Medical Sciences and Research (Deemed to be University), Ambala, IND; 2 Physiology, Dr. N Y Tasgaonkar Institute of Medical Science and Raigad Hospital and Research Centre, Karjat, IND; 3 Musculoskeletal Physiotherapy, Dr. N Y Tasgaonkar College of Physiotherapy, Karjat, IND; 4 Physiology, Naraina Medical College and Research Centre, Kanpur, IND

**Keywords:** autonomic nervous system (ans), gut-brain-immune axis, gut microbiota dysbiosis, heart rate variability, infectious diseases, inflammation, microbiome, neuroimmune interaction, vagus nerve

## Abstract

The human gastrointestinal tract harbors a complex microbial ecosystem that plays a fundamental role in maintaining immune balance and defending against infectious diseases. Disruption of this ecosystem, known as dysbiosis, has been increasingly associated with impaired immune responses and heightened susceptibility to infections. Emerging evidence highlights the importance of the gut-brain-immune axis, particularly the role of the autonomic nervous system (ANS), in regulating these interactions. This systematic review aimed to examine the relationship between gut microbiota dysbiosis and infectious diseases, with specific emphasis on the influence of autonomic dysfunction on disease susceptibility and outcomes. A comprehensive search of PubMed/Medical Literature Analysis and Retrieval System Online (MEDLINE), Scopus, and Google Scholar was conducted to identify relevant observational studies published between 2000 and 2025. Data extraction and quality assessment were performed using standardized tools, including the Newcastle-Ottawa Scale (NOS). A total of 11 studies were included in the qualitative synthesis. The included studies consistently demonstrated that dysbiosis is associated with reduced microbial diversity and loss of beneficial taxa, which in turn were linked to increased infection risk and disease severity. Additionally, autonomic imbalance, reflected by increased sympathetic activity and reduced parasympathetic (vagal) tone, was frequently associated with impaired immune regulation. Reduced heart rate variability was commonly reported, suggesting a potential link between microbial alterations and neuroimmune dysfunction. Overall, the findings suggest that gut microbiota dysbiosis may be associated with infectious disease outcomes, with ANS dysfunction acting as a potential modulatory pathway. These findings should be interpreted with caution, as the included studies are predominantly observational and mechanistic. The results are therefore hypothesis-generating rather than practice-changing, and further high-quality studies are required to establish causal relationships.

## Introduction and background

The gut microbiota constitutes a highly complex and diverse community of microorganisms residing within the human gastrointestinal tract, playing a pivotal role in maintaining physiological homeostasis. These microbial populations are essential for digestion, metabolic regulation, immune modulation, and protection against pathogenic organisms [1-2]. Increasing evidence indicates that alterations in gut microbial composition can significantly influence human health and disease, with disruptions in microbial equilibrium implicated in a wide range of pathological conditions, including infectious diseases [3-4].

The concept of the gut-brain axis has provided valuable insights into the bidirectional communication between the gastrointestinal system and the central nervous system. This interaction encompasses a coordinated network of neural, hormonal, immune, and microbial pathways that collectively regulate host physiology. Expanding on this framework, the gut-brain-immune axis has emerged as a key model for understanding how microbial alterations influence systemic immune responses and disease susceptibility [5-7]. Within this axis, the autonomic nervous system (ANS) plays a central regulatory role by modulating gastrointestinal function, inflammatory responses, and neuroimmune signaling [8,9].

Dysbiosis, defined as an imbalance in the composition and function of the gut microbiota, is increasingly recognized as a major contributor to disease pathogenesis. It is characterized by reduced microbial diversity, depletion of beneficial commensal organisms, and expansion of opportunistic pathogens [8,9]. These alterations can compromise intestinal barrier integrity, resulting in increased permeability and translocation of microbial products into the systemic circulation. Consequently, this process triggers inflammatory cascades and disrupts immune homeostasis, thereby increasing susceptibility to infectious diseases and exacerbating disease severity [10].

The interaction between gut microbiota and the ANS represents a critical mechanistic pathway in this context. The ANS, comprising sympathetic and parasympathetic divisions, exerts significant influence on immune regulation. Increased sympathetic activity is associated with pro-inflammatory responses, whereas parasympathetic activation, primarily mediated through the vagus nerve, facilitates anti-inflammatory effects via the cholinergic anti-inflammatory pathway [8,9]. Dysbiosis has been shown to disrupt this autonomic balance, leading to sympathetic dominance and reduced vagal tone, which may impair immune regulation and increase infection risk [8,9].

Emerging research has further highlighted the role of microbial metabolites, particularly short-chain fatty acids, in modulating neural and immune functions. These metabolites influence intestinal barrier integrity, regulate inflammatory pathways, and interact with neural signaling mechanisms. Additionally, alterations in microbial composition have been linked to changes in neuroimmune communication, suggesting that gut microbiota plays an integral role in shaping systemic responses to infections [8-10].

Advancements in metagenomic sequencing and high-throughput analytical techniques have enabled comprehensive characterization of gut microbial communities and their functional profiles. These technologies have provided critical insights into the relationship between microbiota composition and disease states, facilitating the identification of specific microbial patterns associated with increased infection risk and adverse clinical outcomes [11].

The growing recognition of the gut microbiota’s role in disease has stimulated interest in therapeutic strategies aimed at restoring microbial balance. Interventions such as probiotics, prebiotics, and dietary modifications have demonstrated potential in modulating gut microbiota composition and enhancing host immune responses. Moreover, strategies targeting autonomic regulation, including stress reduction and vagal modulation, are being explored as complementary approaches to improve immune function and reduce disease burden.

Despite these advances, the precise mechanisms underlying the interaction between gut microbiota dysbiosis, ANS dysfunction, and infectious diseases remain incompletely understood. Further research is required to elucidate these complex relationships and to translate emerging evidence into effective clinical interventions. Therefore, this systematic review aims to qualitatively synthesize the available evidence on the association between gut microbiota dysbiosis and infectious disease susceptibility and outcomes, with a specific focus on evaluating whether ANS dysfunction may act as a mediating pathway within the gut-brain-immune axis.

## Review

Methods

This systematic review was carried out following the Preferred Reporting Items for Systematic Reviews and Meta-Analyses (PRISMA) 2020 guidelines and was consistent with the PRISMA-Protocols (PRISMA-P) recommendations for protocol development. The research question was structured using the Population, Intervention, Control, and Outcomes (PICO) framework, with emphasis on the contribution of gut microbiota dysbiosis in infectious diseases and its interaction with the gut-brain-immune axis, particularly via modulation of the ANS [12].

Literature Search

A systematic and comprehensive search strategy was implemented across multiple electronic databases, including PubMed/Medical Literature Analysis and Retrieval System Online (MEDLINE), Scopus, Web of Science, EBSCO Host, Cochrane Controlled Register of Trials (CENTRAL), Cumulative Index to Nursing and Allied Health Literature (CINAHL), Excerpta Medica database (EMBASE), and Google Scholar. Literature published from the inception of each database between 2000 and 2025 was considered eligible.

The search strategy incorporated combinations of keywords and Boolean operators such as: (“gut-brain axis” OR “microbiota-gut-brain axis”) AND (microbiota OR microbiome OR dysbiosis) AND (“autonomic nervous system” OR vagus nerve) AND (infection OR infectious diseases OR sepsis) AND (immune OR inflammation). Detailed search strategy is presented in Appendix A.

To reduce publication bias, grey literature sources, including OpenGrey, WorldCat, ClinicalTrials.gov, World Health Organization International Clinical Trials Registry Platform (WHO ICTRP), and International Standard Randomised Controlled Trial Number (ISRCTN) Registry, were also explored. Additionally, forward citation tracking and manual screening of reference lists were conducted to identify further relevant studies [13-15].

Inclusion and Exclusion Criteria

Studies were included if they investigated the association between gut microbiota and infectious diseases, with consideration of ANS involvement where available; however, studies without direct ANS assessment were also included if they provided relevant mechanistic or clinical insights related to the gut-brain-immune axis. Both human observational studies and experimental studies (animal and in vitro) were included to provide complementary clinical and mechanistic insights.

Given the methodological heterogeneity, studies were categorized and analyzed separately as 1) Human clinical/observational studies, and 2) Experimental studies (animal and in vitro models). No quantitative pooling across these categories was performed, and findings were synthesized qualitatively.

Studies were excluded if they focused on non-infectious conditions or did not assess gut microbiota or autonomic nervous system parameters. Additionally, review articles, editorials, conference abstracts, and duplicate studies were excluded. 

Data Extraction

Data extraction was performed independently by two reviewers using a standardized and pre-defined extraction form. Extracted data included study design, demographic characteristics, microbiota-related parameters (e.g., alpha diversity and taxa abundance), autonomic indicators such as heart rate variability and vagal tone, immune biomarkers including IL-6, TNF-α, and IFN-γ, and clinical outcomes such as disease severity and mortality. Any discrepancies between reviewers were resolved through discussion until consensus was achieved.

Quality Assessment

The methodological quality and risk of bias of included studies were assessed using the Newcastle-Ottawa Scale (NOS), which evaluates study selection, comparability, and outcome assessment. Two reviewers conducted the assessment independently, and disagreements were resolved through consensus [16].

Certainty of Evidence

The certainty of evidence was evaluated using the Grading of Recommendations Assessment, Development, and Evaluation (GRADE) framework, which considers factors such as risk of bias, inconsistency, indirectness, imprecision, and publication bias. Based on these domains, the quality of evidence was categorized as high, moderate, low, or very low, ensuring transparency in the interpretation of findings [17].

Data Synthesis

Due to considerable heterogeneity across study designs, populations, microbiota assessment techniques, and outcome measures, quantitative meta-analysis was not feasible. Therefore, a qualitative synthesis approach was adopted. Findings were systematically organized into thematic domains, including gut microbiota dysbiosis, autonomic nervous system involvement, and immune interactions in infectious diseases. Within each domain, patterns, mechanisms, and relationships were identified and synthesized to provide an integrated understanding of the gut-brain-immune axis.

Ethical Considerations and Registration

As this review utilized previously published data, ethical approval and informed consent were not required. The study adhered to PRISMA 2020 guidelines to maintain methodological transparency and rigor. The review protocol was prospectively registered with the International Prospective Register of Systematic Reviews (PROSPERO; Registration ID: CRD420261360051) to ensure methodological transparency, enhance reproducibility, and minimize potential reporting bias.

Results

Study Identification

A total of 1,320 records were identified through database searching, with an additional 35 records identified from other sources. After removal of duplicates (n = 150), records excluded by automation tools (n = 59), and records removed for other reasons (n = 61), a total of 1,050 records remained for screening. Of these, 301 records were excluded based on title and abstract screening.

A total of 749 reports were sought for retrieval, of which 443 could not be retrieved. Subsequently, 306 full-text articles were assessed for eligibility. Among these, 295 articles were excluded as they did not assess gut microbiota or microbiome-related outcomes (n = 145), did not evaluate autonomic nervous system function or related parameters (n = 112), and were not related to infectious diseases or infection outcomes (n = 38). From other sources, 35 records were identified, of which 31 reports could not be retrieved. The remaining four reports were assessed for eligibility, and all four were excluded (no microbiota/infectious condition relevance, n = 2; abstract only/not available, n = 2).

Characteristics of the Included Studies

The included studies comprised a combination of human observational studies and experimental investigations, including animal and in vitro models. Human studies primarily evaluated clinical associations, whereas experimental studies provided mechanistic insights into the gut-brain-immune axis. Most studies assessed gut microbiota composition using 16S rRNA sequencing or metagenomic analysis. Immune responses were commonly evaluated through cytokine profiling (e.g., IL-6, TNF-α, IFN-γ), while autonomic function was measured using indicators such as vagal activity and heart rate variability. Several studies focused on infectious conditions, including COVID-19, sepsis, and systemic inflammatory states.

Qualitative Data Synthesis

Given the heterogeneity in study methodologies and outcome measures, a qualitative synthesis was undertaken. Findings were categorized into three primary thematic areas: gut microbiota dysbiosis, autonomic nervous system involvement, and immune system interactions. This classification enabled identification of consistent trends and mechanistic links between the gut-brain-immune axis and infectious disease outcomes.

Results of the Search

**Figure 1:**
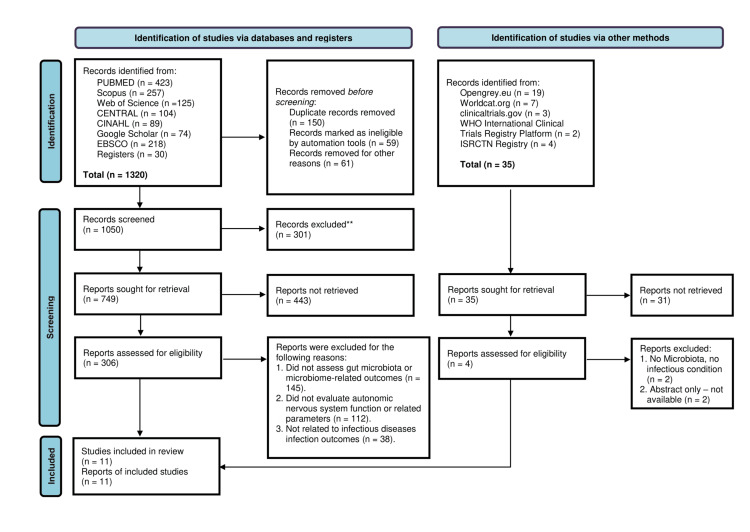
PRISMA flow diagram of the study selection process. PRISMA: Preferred Reporting Items for Systematic Reviews and Meta-Analyses; CENTRAL: Cochrane Controlled Register of Trials; CINAHL: Cumulative Index to Nursing and Allied Health Literature; ISRCTN Registry: International Standard Randomised Controlled Trial Number Registry The diagram was generated using the PRISMA 2020 Flow Diagram web application developed by Haddaway NR, McGuinness LA, and colleagues (United Kingdom).

The PRISMA-guided selection process resulted in 11 studies being included in the final analysis in Figure 1. These studies encompassed observational and mechanistic designs examining microbiota composition, autonomic regulation, and immune responses [3,6,7,8,11,18-23]. 

Methodological Quality and Risk of Bias

The methodological quality of the included studies was assessed using the NOS (Table 1). Overall, the studies demonstrated moderate to high quality, with scores ranging from 5 to 9. High-quality studies, including those by Schirmer et al. [6], Haak et al. [7], Zuo et al. [11], Erny et al. [19], and Buffie et al. [20] showed strong study design, appropriate participant selection, and reliable outcome assessment.

**Table 1:** Quality assessment of the included studies using the Newcastle–Ottawa Scale. Higher scores indicate better methodological quality across selection, comparability, and outcome domains.

Study	Selection (0–4)	Comparability (0–2)	Outcome (0–3)	Total	Quality
Zuo et al. [11]	4	2	3	9	High
Haak et al. [7]	3	2	3	8	High
Schubert et al. [21]	3	1	2	6	Moderate
Buffie et al. [20]	3	1	3	7	High
Erny et al. [19]	3	1	3	7	High
Thaiss et al. [3]	3	1	2	6	Moderate
Tracey et al. [8]	2	1	2	5	Moderate
Borovikova et al. [18]	2	1	2	5	Moderate
Wypych et al. [22]	3	1	2	6	Moderate
Dantzer et al. [23]	2	1	2	5	Moderate
Schirmer et al. [6]	3	2	3	8	High

Overall, the methodological quality of included studies ranged from moderate to high; however, several studies had NOS scores in the lower range (5-6), indicating potential methodological limitations. Therefore, the findings should be interpreted with caution, particularly in light of study heterogeneity and limited control for confounding factors. Variability in outcome measures and inconsistent reporting of autonomic parameters were also observed, which should be considered when interpreting the findings. 

Certainty of Evidence

Overall, the certainty of evidence was judged to be low to moderate across outcomes. Observational studies were initially rated as low certainty according to the GRADE framework and were considered for upgrading only where consistent findings, plausible biological mechanisms, and strength of association were observed. However, due to heterogeneity in study designs, limited control for confounding, and reliance on mechanistic evidence, the overall certainty remained predominantly low to moderate, as summarized in Table 2. Evidence linking gut microbiota and immune responses was rated as high certainty due to consistency and low risk of bias.

**Table 2:** Certainty of evidence assessment summary The summary of certainty of evidence was assessed using the Grading of Recommendations Assessment, Development, and Evaluation (GRADE) framework across key outcomes. Certainty levels reflect overall confidence in the evidence based on methodological quality and consistency.

Outcome	Evidence Type	Risk of Bias	Consistency	Certainty	Key References
Microbiota dysbiosis	Observational	Moderate	High	Moderate	[6,7,11,21]
Immune response	Experimental	Low	High	High	[3,19,20]
Autonomic nervous system involvement	Mechanistic	Moderate	Moderate	Moderate	[8,18,23]
Infection severity	Clinical	Moderate	High	Moderate	[7,11]

Certainty of evidence was assessed using the GRADE approach. Observational studies were initially classified as low certainty and were evaluated for potential upgrading based on factors such as consistency of findings, strength of associations, and biological plausibility. No formal upgrading was applied in the absence of strong, consistent, and high-magnitude effects. Downgrading was considered where there was risk of bias, heterogeneity, and indirectness of evidence.

In contrast, evidence related to ANS involvement was graded as moderate, primarily due to heterogeneity and reliance on indirect measures such as heart rate variability. Similarly, evidence connecting dysbiosis with infection severity was considered moderate due to variability in study populations and designs.

The certainty of evidence assessment (Table 2) demonstrated variability across outcomes depending on study design and methodological quality. Evidence related to microbiota dysbiosis, derived from observational studies, showed a moderate risk of bias but high consistency, resulting in an overall moderate level of certainty. In contrast, evidence on immune response, primarily based on experimental studies, exhibited low risk of bias and high consistency, yielding a high level of certainty. Findings regarding ANS involvement, supported by mechanistic studies, indicated moderate risk of bias and moderate consistency, leading to moderate certainty. Similarly, evidence addressing infection severity from clinical studies showed moderate risk of bias with high consistency, resulting in a moderate level of certainty. Overall, as summarized in Table 2, the certainty of evidence ranged from moderate to high, with the strongest support observed for immune response outcomes.

Synthesis of Key Findings

Gut microbiota dysbiosis: All included studies reported notable alterations in gut microbiota composition, including reduced beneficial bacteria, increased pathogenic organisms, and disrupted metabolic activity. Dysbiosis was associated with increased intestinal permeability and systemic inflammation, contributing to higher susceptibility to infections.

ANS involvement: Evidence regarding the role of the ANS as a mediator linking gut microbiota and immune responses was limited. A small number of studies suggested that microbial signals may influence neural pathways, particularly via vagal nerve activity. Some studies reported associations between autonomic imbalance, which is characterized by increased sympathetic activity and reduced parasympathetic tone, and impaired immune regulation. Reduced heart rate variability was also described as a potential indirect marker of autonomic dysfunction. However, these findings were derived from a limited subset of studies and should be interpreted with caution.

Immune system interactions: A consistent observation across studies was the link between dysbiosis and immune activation. Elevated levels of pro-inflammatory cytokines, including IL-6, TNF-α, and IFN-γ, were reported. Additionally, microbial metabolites were shown to modulate immune cell activity and systemic inflammation.

Integration of mechanisms and clinical outcomes: The findings indicate that gut microbiota dysbiosis contributes to immune dysregulation through multiple pathways, including cytokine signaling, microbial metabolite activity, and neuroimmune interactions, as depicted in Table 3. These mechanisms collectively impact clinical outcomes, such as increased infection severity, compromised immune defense, and higher mortality in critically ill patients.

**Table 3:** Summary of included studies with mechanisms and outcomes Summary of key studies highlighting the relationship between gut microbiota alterations, autonomic–immune mechanisms, and infectious disease outcomes. This table describes exposures, underlying mechanisms, and their clinical implications. CNS: central nervous system

Study	Intervention/Exposure	Mechanism	Outcome
Zuo et al. [11]	Gut microbiota dysbiosis	Immune modulation and cytokine activation	Increased disease severity
Buffie et al. [20]	Microbiota restoration	Metabolite-mediated immune protection	Improved infection resistance
Erny et al. [19]	Germ-free model	Microglial dysfunction and impaired immune maturation	Reduced CNS immunity
Schirmer et al. [6]	Microbiome variation	Cytokine modulation	Altered immune response variability
Haak et al. [7]	ICU-associated microbiota changes	Dysbiosis and systemic inflammation	Increased mortality

Summary of Key Results

Overall, the evidence supports a bidirectional relationship between gut microbiota, the ANS, and immune responses in infectious diseases. Dysbiosis is consistently associated with altered immune function and greater disease severity. The autonomic nervous system, particularly vagal pathways, plays a crucial role in regulating inflammation, while microbial metabolites influence both neural and immune mechanisms. Together, these interactions significantly affect susceptibility, progression, and outcomes of infectious diseases, forming the basis for further discussion of mechanistic and clinical implications.

A detailed summary of study characteristics, including design, population, microbiota assessment, autonomic parameters, and key findings, is presented in Table 4

**Table 4:** Characteristics of included studies evaluating gut microbiota, ANS involvement, and infectious disease outcomes. ANS: autonomic nervous system; CNS: central nervous system

Study	Study Design	Population	Microbiota Assessment	ANS Assessment	Key Findings	Reference
Zuo et al.	Observational cohort	Patients with COVID-19	16S rRNA sequencing	Not directly assessed	Reduced microbial diversity and altered composition associated with increased disease severity	[11]
Haak et al.	Observational	ICU patients with sepsis	Microbiome profiling	Indirect (inflammatory markers)	Microbiota alterations linked to infection outcomes and increased mortality	[7]
Schubert et al.	Case-control	Patients with *Clostridium difficile* infection	Microbiome analysis	Not directly assessed	Distinct microbial profiles differentiate infected patients from healthy controls	[21]
Buffie et al.	Experimental	Infection models	Microbiota restoration studies	Not directly assessed	Restoration of microbiota enhances resistance to infection	[20]
Erny et al.	Experimental	Germ-free models	Microbiome manipulation	CNS–immune interaction	Microbiota regulates immune maturation and microglial function	[19]
Thaiss et al.	Mechanistic	Human and experimental studies	Microbiome–immune interaction	Not directly assessed	Microbiota regulates innate immune responses and inflammatory pathways	[3]
Tracey	Mechanistic	Experimental models	Not applicable	Vagus nerve signaling	Cholinergic anti-inflammatory pathway modulates immune responses	[8]
Borovikova et al.	Experimental	Animal and human models	Not applicable	Vagal stimulation	Vagus nerve stimulation attenuates systemic inflammatory responses	[18]
Wypych et al.	Mechanistic review	Respiratory disease context	Microbiome studies	Not directly assessed	Microbiota influences immune responses in respiratory infections	[22]
Dantzer	Mechanistic	Neuroimmune studies	Not applicable	Neuroimmune pathways	Bidirectional interaction between the brain and immune system influences disease processes	[23]
Schirmer et al.	Observational	Human cohort	Microbiome and cytokine profiling	Not directly assessed	Microbiota influences cytokine production and inter-individual immune variability	[6]

Discussion

This systematic review synthesizes current evidence on the association between gut microbiota dysbiosis and infectious diseases, with particular emphasis on the role of the gut-brain-immune axis and ANS modulation. The findings demonstrate a consistent relationship between alterations in gut microbial composition, immune dysregulation, and adverse infectious outcomes. Across the included studies, moderate to high methodological quality strengthens the reliability of these observations, although certain limitations must be considered when interpreting the results [1-4].

Gut microbiota dysbiosis emerged as a central factor influencing susceptibility to infectious diseases. Reduced microbial diversity and depletion of beneficial commensal organisms were consistently associated with increased intestinal permeability and systemic inflammation. These alterations facilitate the translocation of microbial products into the circulation, triggering inflammatory cascades and impairing immune homeostasis. The observed elevation in pro-inflammatory cytokines further supports the role of dysbiosis in promoting both infection risk and disease severity [7,11].

A key contribution of this review is the identification of the ANS as a critical intermediary within the gut-brain-immune axis. Evidence suggests that gut microbiota communicates with the central nervous system through neural pathways, particularly via vagus nerve signaling. Dysbiosis appears to disrupt this bidirectional communication, leading to autonomic imbalance characterized by increased sympathetic activity and reduced parasympathetic (vagal) tone. This imbalance is associated with impaired immune regulation and exaggerated inflammatory responses, thereby contributing to poorer infectious outcomes [8-10]. Therefore, while the ANS represents a plausible mechanistic link, it cannot be considered a definitive or primary pathway based on the current evidence base. However, some studies reported variability in microbiota composition across different populations, suggesting that host factors and environmental influences may also play a significant role.

The mechanistic basis of these interactions can be explained through integrated neural, humoral, and microbial pathways. Neural signaling via the autonomic nervous system enables communication between the gut and brain, while circulating mediators such as cytokines, hormones, and microbial metabolites regulate systemic immune responses. Microbial-derived metabolites, including short-chain fatty acids, play a crucial role in maintaining intestinal barrier integrity and modulating inflammatory pathways. Disruption of these interconnected systems in the setting of dysbiosis may lead to impaired neuroimmune communication and progression of disease [3,6,9].

Autonomic dysfunction may represent an important component of the gut-brain-immune interaction. Available evidence suggests that reduced vagal tone and increased sympathetic activity are associated with heightened inflammatory responses and altered immune homeostasis. Heart rate variability, a commonly used non-invasive surrogate marker of autonomic function, has frequently been reported as reduced in individuals with infectious diseases, indicating a potential state of neuroimmune imbalance. The cholinergic anti-inflammatory pathway, mediated through vagal signaling, has been proposed as a mechanism linking neural and immune responses. However, it is important to note that much of the current evidence is based on indirect measures and observational or experimental studies. Therefore, autonomic involvement should be considered a plausible or hypothesized pathway rather than a confirmed mechanism [8,9,22,23].

From a clinical perspective, these findings suggest that gut microbiota composition may serve as a potential biomarker for identifying individuals at increased risk of infections, while autonomic markers such as heart rate variability may provide additional prognostic information. However, the current evidence is primarily observational, and the clinical utility of these markers requires further validation in prospective studies. Although modulation of gut microbiota and autonomic function has been proposed as a potential therapeutic approach in other contexts, such strategies were not directly evaluated in the studies included in this review and therefore remain beyond the scope of the present analysis [9,10].

This review has several strengths, including adherence to PRISMA guidelines, comprehensive literature search, and systematic quality assessment using validated tools such as the NOS and GRADE framework. The integration of microbiota, autonomic, and immune components provides a more comprehensive understanding of the gut-brain-immune axis in infectious diseases. Future research should focus on well-designed longitudinal and interventional studies to better clarify the relationships between gut microbiota dysbiosis, autonomic dysfunction, and infectious disease outcomes. Standardization of microbiota assessment techniques and autonomic function measures will further enhance comparability across studies. Advances in microbiome science and precision medicine may facilitate the development of individualized therapeutic strategies targeting both microbial composition and neuroimmune regulation. Further exploration of microbiota-based interventions and autonomic modulation holds promise for improving prevention and management strategies in infectious diseases [10,22,23].

Limitations

This systematic review has several limitations that should be considered when interpreting the findings. First, the included studies demonstrated substantial heterogeneity in study design, population characteristics, microbiota assessment methods, and outcome measures, which precluded quantitative meta-analysis. Second, most studies were observational in nature, limiting the ability to establish causal relationships between gut microbiota dysbiosis, autonomic dysfunction, and infectious disease outcomes. Third, direct assessment of ANS function was limited, with many studies relying on indirect measures such as heart rate variability. Additionally, this review included a combination of human, animal, and mechanistic studies. While this approach allowed for a broader understanding of underlying mechanisms, it may limit direct clinical applicability and introduce challenges in translating findings across study types. A substantial proportion of reports (443/749; ~59%) could not be retrieved despite being identified during screening. This introduces a potential risk of selection bias, as unavailable studies may differ systematically from those included. Furthermore, variability in microbiome sequencing techniques and a lack of standardized methodologies may affect comparability across studies. Finally, potential publication bias and restriction to English-language articles may have influenced the overall evidence base.

## Conclusions

This systematic review highlights a consistent association between gut microbiota dysbiosis and infectious disease outcomes, with evidence suggesting interactions within the gut-brain-immune axis. Alterations in microbial composition were commonly associated with markers of systemic inflammation and immune dysregulation, as well as features of impaired barrier function. Evidence also suggests that ANS activity may represent a plausible intermediary pathway linking gut microbiota and immune responses. Patterns of autonomic imbalance characterized by increased sympathetic activity, reduced vagal tone, and lower heart rate variability were frequently observed alongside inflammatory and adverse clinical profiles. However, these observations are largely derived from indirect measures and predominantly observational or preclinical studies; therefore, causality cannot be established. While these findings provide valuable mechanistic insights, their direct clinical applicability remains limited. Future research should prioritize well-designed prospective and interventional studies to clarify causal pathways and determine the translational relevance of targeting the microbiota-autonomic-immune axis.

## Appendices

Appendix A

Detailed Search Strategy

Title of Review: The Gut-Brain-Immune Axis: Role of Microbiota Dysbiosis and Autonomic Nervous System in Infectious Diseases

Databases Searched: PubMed, Scopus, Web of Science, CENTRAL (Cochrane Central Register of Controlled Trials), CINAHL, Google Scholar, EBSCOhost, Clinical Trial Registers

Search Period: Literature published from the inception of each database between 2000 and 2025 was considered eligible

1. PubMed Search Strategy

("gut-Brain Axis"[Mesh] OR "gut brain axis" OR "brain gut axis" OR "microbiota-gut-brain axis")

AND

("microbiota"[Mesh] OR "microbiome" OR "gut microbiota" OR "dysbiosis")

AND

("autonomic Nervous System"[Mesh] OR "autonomic nervous system" OR "ANS" OR "vagus nerve" OR "sympathetic nervous system" OR "parasympathetic nervous system")

AND

("infectious Disease"[Mesh] OR "infection" OR "infectious diseases")

AND

("immune System"[Mesh] OR "immune response" OR "immunity")

Filters applied: Humans, English language, Publication years 2000-2025

2. Scopus Search Strategy

(TITLE-ABS-KEY ("gut brain axis" OR "brain gut axis" OR "microbiota gut brain axis"))

AND TITLE-ABS-KEY ("microbiota" OR "microbiome" OR "dysbiosis"))

AND TITLE-ABS-KEY ("autonomic nervous system" OR "ANS" OR "vagus nerve"))

AND TITLE-ABS-KEY ("infection" OR "infectious disease"))

AND TITLE-ABS-KEY ("immune" OR "immunity"))

AND (PUBYEAR >1999 AND PUBYEAR <2026)

Limits: English language

3. Web of Science Search Strategy

TS = (("gut brain axis" OR "brain gut axis" OR "microbiota gut brain axis")

AND ("microbiota" OR "microbiome" OR "dysbiosis")

AND ("autonomic nervous system" OR "ANS" OR "vagus nerve")

AND ("infection" OR "infectious disease")

AND ("immune" OR "immunity"))

Refined by: English Language

Timespan: 2000-2025

4. CENTRAL (Cochrane Library)

("gut brain axis" OR "microbiota gut brain axis")

AND ("microbiota" OR "microbiome" OR "dysbiosis")

AND ("autonomic nervous system" OR "vagus nerve")

AND ("infection" OR "infectious disease")

AND ("immune response" OR "immunity")

5. CINAHL (via EBSCOhost)

(MH "Gut-Brain Axis" OR "gut brain axis")

AND (MH "Microbiota" OR "microbiome" OR "dysbiosis")

AND (MH "Autonomic Nervous System" OR "ANS" OR "vagus nerve")

AND (MH "Infectious Diseases" OR "infection")

AND (MH "Immune System" OR "immune response")

Limiters: English, 2000-2025

6. Google Scholar Search Strategy

"gut brain axis" AND microbiota AND dysbiosis AND "autonomic nervous system" AND infection AND immunity

Screening was limited to the first 200 most relevant results sorted by relevance.

7. EBSCOhost (General Databases)

("gut brain axis" OR "microbiota gut brain axis")

AND ("microbiota" OR "microbiome" OR "dysbiosis")

AND ("autonomic nervous system" OR "vagus nerve")

AND ("infection" OR "infectious disease")

AND ("immune system" OR "immunity")

Filters: English, 2000-2025

8. Clinical Trial Registers

Sources: ClinicalTrials.gov, WHO ICTRP

Search terms:

"gut brain axis" OR "microbiota" OR "dysbiosis" AND "infection" AND "autonomic nervous system"
